# Development and optimization of a glycerol-based fed-batch strategy for the production of peroxidases with *Komagataella phaffii* under the P_DF_ promoter

**DOI:** 10.1186/s12934-026-03055-1

**Published:** 2026-07-10

**Authors:** Christian Mathis Wagner, Katharina Schwartz, Hannah Czech, Ines Lamping, Xavier Garcia-Ortega, Anton Glieder, Wolfgang Wiechert, Marco Oldiges

**Affiliations:** 1 Institute of Bio- and Geosciences IBG-1: Biotechnology, Forschungszentrum Jülich GmbH, 52425 Jülich, Germany; 2 Institute of Biotechnology, RWTH Aachen University, Worringerweg, 52074 Aachen, Germany; 3 bisy GmbH, 8200 Hofstätten an Der Raab, Austria; 4 Computational Systems Biotechnology (AVT.CSB), RWTH Aachen University, 52074 Aachen, Germany

**Keywords:** Bioprocess optimization, Crude glycerol, Glycerol derepression, P_DF_ promoter, *Komagataella phaffii*, *Pichia pastoris*, Peroxidase, Secretion

## Abstract

**Background:**

Unsaturated polyester resins are commonly polymerized using cobalt-based siccatives. However, growing health concerns and increasing demand for cobalt highlight the need for more sustainable alternatives. Heterologously produced peroxidases, produced through secretory expression in *Komagataella phaffii* (*K. phaffii*, previously classified as* Pichi a pastoris*), represent a promising substitute for cobalt in such applications. To tailor the bioprocess for the production of peroxidases, dedicated bioprocess development and optimization are required. Rather than relying on methanol induction, the process should aim to achieve production via glycerol derepression under the control of the P_DF_ promoter. This strategy mitigates the safety risks and metabolic stress associated with methanol utilization.

**Results:**

This study presents the development and optimization of a three-phase bioprocess for peroxidase production in *K. phaffii* under the P_DF_ promoter, induced via glycerol derepression. The process comprises a batch growth phase, a growth and induction fed-batch phase and a second fed-batch phase for product formation. Exponential feeding at 50% of the maximum specific growth rate (µ_max_) was identified as the optimum, balancing growth, induction and productivity. During the production phase, a glycerol feed at 20% of the maximum uptake rate (q_Smax_) and a duration of up to 96 h yielded the best results. The final optimized process achieved a titer of 12.36 U*mL^−1^, a yield of 46.31 U*g^−1^, and a space–time yield of 0.081 U*mL^−1^*h^−1^, which clearly outcompeted the methanol reference process by a factor of 13.4. Strikingly, the use of crude glycerol further improved performance by approximately 10%, demonstrating process robustness. Overall, this bioprocess enables efficient and sustainable peroxidase production, offering a viable alternative to cobalt-based catalysts in polyester resin curing.

**Conclusions:**

This work demonstrates the feasibility of a robust, methanol-free bioprocess for peroxidase production in *K. phaffii*, driven by glycerol derepression under the P_DF_ promoter. The optimized process achieves high titers and yields, maintains performance even with crude glycerol, and could support sustainable replacement of cobalt-based siccatives in industrial polymerization. These findings provide a valuable foundation for advancing biocatalytic solutions in green manufacturing.

## Background

Unsaturated polyester resins are commonly used in the manufacturing process of composite materials for applications ranging from wind turbine blades and boat hulls to other structural components. The polymerization or “curing” of these resins is typically started by cobalt-based siccatives [1]. In this process, cobalt carboxylates or related compounds start the reaction of peroxides, initiating radical chain polymerization that leads to the hardening of the resin matrix [2]. Global cobalt demand reached approximately 200 kilotons in 2023, more than doubling since 2016, reflecting its increasing use across diverse industrial sectors [3]. Despite their robust utilization, cobalt-based siccatives present serious drawbacks, including their classification as carcinogenic, the poor environmental conditions associated with cobalt mining, and growing competition for cobalt in other technologies such as batteries, superalloys, and permanent magnets [4–7].

To overcome these limitations, there is growing interest in developing sustainable and non-hazardous alternatives. Protein-based catalysts represent a promising solution, offering advantages such as biodegradability, substrate specificity, and high catalytic efficiency [4, 8, 9]. Among these, peroxidases have emerged as viable substitutes due to their ability to catalyze reactions analogous to those initiated by cobalt compounds during resin curing [10]. In particular, fungal peroxidases are highly versatile biocatalysts [11, 12]. In addition to their natural roles in lignocellulose degradation and the transformation of humic substances, they are applied in fields such as analytical chemistry and industrial wastewater treatment [13, 14]. In this study, an unspecific peroxygenase from *Hypoxylon* sp. EC38 (*Hsp*UPO) was selected as the biocatalyst for resin polymerization [15].

*Komagataella phaffii *(*K. phaffii*, previously classified as *Pichia pastoris*) was chosen as the heterologous host for secretory peroxidase production [15]. This methylotrophic yeast is well-established in the large-scale production of recombinant proteins for applications including food additives, detergents, and wastewater treatment [16, 17]. Its suitability for high-cell-density cultivation, coupled with its available tools for genetic engineering, has been extensively demonstrated [16, 18, 19]. Furthermore, *K. phaffii* can secrete heterologous proteins efficiently into the cultivation medium, with minimal endogenous secretion, thus simplifying downstream processing [17, 20].

One of the key advantages of *K. phaffii* is the availability of a diverse set of strongly inducible promoters for high-level protein expression [16, 21–23]. In this study, the versatile and tunable P_DF_ promoter was utilized, since in early studies classical methanol inducible (P_*AOX1*_) and constitutive (P_GAP_) promoters delivered only very low enzyme titer. This promoter, a fragment derived from the *Hansenula polymorpha*, P_FMD_ promoter is characterized by its dual-mode induction: the native sequence version of this strong promoter can be activated not only by methanol but also through glycerol derepression, i.e., under conditions of limited glycerol feeding. Expression remains repressed during growth on glucose or glycerol and is strongly activated only after depletion of the repressing carbon source. This enables a practical two-phase cultivation strategy, with biomass accumulation followed by recombinant protein production. This feature offers enhanced flexibility in process design and provides an opportunity to reduce or eliminate methanol-dependent induction, even though, in some specific scenarios, the expression can also be further induced by methanol [19, 24–26].

Traditionally, methanol has served as the main inducer in *K. phaffii* expression systems, particularly through the activation of the P_*AOX1*_ promoter. Its tightly regulated induction and the separation of growth and production phases enable precise control over recombinant protein expression [27–29]. However, methanol poses safety and scalability challenges due to its flammability, toxicity, and high oxygen demand during metabolization [30, 31]. In contrast, glycerol is a non-toxic, non-flammable carbon source that can support high biomass accumulation and, when used with promoters which become active under carbon-limited cultivation conditions like P_DF_, could also drive high levels of protein expression [24, 26]. In general, glycerol-based processes can be attributed to being safer and more energy-efficient and avoid the formation of compounds with toxic potential, such as formaldehyde and hydrogen peroxide. While methanol remains a widely used inducer, especially if tightly repressible systems are needed, glycerol offers a safer and potentially more scalable alternative [30–32].

In addition to using purified glycerol, this study also explores the application of crude glycerol as a carbon source. As a sidestream of biodiesel production, crude glycerol is available at lower cost and facilitates a more sustainable and circular bioeconomy. It frequently contains impurities such as methanol, fatty acids, and salts, which may support microbial growth or partially substitute for complex media supplements [33–35]. These advantages make crude glycerol viable for high-cell-density cultivations [35, 36]. However, the potential presence of inhibitory substances and intense salt load demand careful process optimization to ensure robustness and reproducibility.

The implementation of the P_DF_ promoter introduces new opportunities but also new challenges for bioprocess development. Unlike tightly regulated methanol-based systems, derepression-based systems require fine-tuned control of glycerol feeding strategies to balance microbial growth and protein production. Moreover, the utilization of crude glycerol introduces additional variables that influence metabolic activity, protein expression, and overall process performance. Consequently, bioprocess development is essential to fully characterize and exploit the potential of this alternative promoter system for the production of peroxidase suitable for industrial polymer applications.

Here, we report the development and optimization of a methanol-free bioprocess for the production of unspecific peroxygenase (*Hsp*UPO) in *K. phaffii*, using glycerol as the sole carbon source. The process strategy consists of an initial batch phase, followed by a two-stage fed-batch approach: a first fed-batch phase for biomass formation and induction, and a second fed-batch phase with lowered constant feeding for production via glycerol derepression. The main growth phase, i.e. the first fed-batch phase, was systematically optimized considering linear vs. exponential feed profiles. For the latter, the specific growth rate corresponding to 50% of the maximum growth rate was found to be the optimal condition. The following production phase was optimized with regard to glycerol feed rate and duration, showing that 20% of the maximum glycerol uptake rate yielded optimal results. The final optimized process achieved enzyme activities of 12.36 U*mL^−1^, 46.31 U*g^−1^ yield, and a space–time yield of 0.081 U*mL^−1^*h^−1^ and substantially outcompeted the methanol-based reference process by a factor of 13.4. Furthermore, the same process using crude glycerol, showed an approximately 10% increase in process performance, stressing the potential of low-cost, carbon sources derived from bio-based sidestreams for sustainable enzyme production.

## Methods

### Reagents and chemicals

All chemicals used in this study were sourced from ROTH (Karlsruhe, Germany) and Merck (Sigma-Aldrich, St. Louis, MO, USA).

### Strain

All experiments used the bisy strain BYS110388, a *K. phaffii* strain based on the proprietary bisy heme jackpot strain BSYBG11JP-HP, transformed with a linearized commercial pBSY5S1Z vector (bisy GmbH, Hofstätten, Austria), containing the gene coding for mature *Hsp*UPO ligated downstream of P_DF_, allowing to express the peroxidase gene either by methanol induction or limited glycerol or glucose feed.

### Cryo conservation

*K. phaffii* was grown in YPG medium (10 g*L^−1^ yeast extract, 20 g*L^−1^ peptone, 20 g*L^−1^ glycerol) using 50 mL in 500 mL Erlenmeyer flasks on an orbital shaker (Infors HT GmbH, Einsbach, Germany) at 250 rpm (25 mm orbital diameter) and 30 °C for 18 h. After measuring the optical density at 600 nm (OD_600_), cultures were centrifuged at 4000 $xg$ and 4 °C for 15 min (Centrifuge CT 15RE, VWR International GmbH, Darmstadt, Germany). The pellets were resuspended in residual supernatant to reach an OD_600_ of 90, then mixed 1:1 with a sterile 500 g*L^−1^ glycerol solution. The suspension was aliquoted into 2 mL cryovials and stored at − 80 °C.

### Seed cultures

Seed cultures were prepared by inoculating 50 mL of YPG medium with 20 µL of thawed cryoconserved culture suspension of *K. phaffii*. Cultivation occurred in 500 mL Erlenmeyer flasks on an orbital shaker (Infors HT GmbH, Einsbach, Germany) at 250 rpm (25 mm orbital diameter) and 30 °C for 20 h.

### Bioreactor cultivations

Main cultivations were performed in a 1.7 L bioreactor system with four glass stirred-tank reactors (STRs), each equipped with two Rushton impellers (DASGIP, Jülich, Germany). Each reactor contained 0.7 L of basal salt batch medium (BSM) consisting of 1.96 g*L^−1^ citric acid, 0.02 g*L^−1^ calcium dichloride dihydrate, 12.6 g*L^−1^ diammonium sulfate, 0.5 g*L^−1^ magnesium sulfate heptahydrate, 0.9 g*L^−1^ potassium chloride, 1 g*L^−1^ dipotassium hydrogen phosphate, 1 g*L^−1^ potassium dihydrogen phosphate, 4*10^–4^ g*L^−1^ biotin, 20 g*L^−1^ glycerol and 4.6 mL*L^−1^ PTM_1_ (0.02 g*L^−1^ boric acid, 0.82 g*L^−1^ cobalt dichloride hexahydrate, 0.08 g*L^−1^ sodium iodide, 0.2 g*L^−1^ disodium molybdate, 6 g*L^−1^ copper sulfate pentahydrate, 65 g*L^−1^ iron sulfate heptahydrate, 3.36 g*L^−1^ manganese sulfate hydrate, 20 g*L^−1^ zinc dichloride and 2.717 mL*L^−1^ sulfuric acid). The cultures were grown in fed-batch mode at 30 °C, with pH maintained at 6.0 using 20% (w*v^−1^) ammonium hydroxide and 4 M phosphoric acid. pH was monitored with 405-DPAS-SC-K8S/225/120 electrodes (Mettler Toledo, Gießen, Germany) and dissolved oxygen (DO) was kept above 30% using VisiFerm DO 225 optodes (Hamilton, Reno, NV, USA). DO was controlled by adjusting the i) stirring speed (400–1500 rpm), ii) gas flow rate (42–84 nL_gas_*L_liquid_*h^−1^) and iii) oxygen concentration in the inlet gas (21–100%) in a cascaded fashion.

The starting OD_600_ for the cultivations was 0.2. After the initial 20 g*L^−1^ glycerol batch phase (~ 18 h), a fed-batch growth and induction phase with biomass accumulation was started, followed by a second fed-batch phase for peroxidase production.

Constant glycerol feed rates were set based on the maximum glycerol uptake rate experimentally determined at the transition to the second fed-batch phase. A pulse of 3 g of glycerol was added to the culture after the completion of the first fed-batch phase, and the time required to metabolize the pulse was determined by monitoring the DO signal. With this information, the maximum glycerol uptake rate in g*h^−1^ was determined. The respective feeding strategies are described in the results section of this manuscript. Samples were taken at regular intervals for product analysis.

### BioLector cultivations

Microscale cultivations were conducted in a BioLectorXT microbioreactor system (Beckman Coulter, Brea, USA) using microfluidic FlowerPlates of the type BOH1 with gas-permeable sealing foils (MTP-MF32-BOH1; Beckman Coulter, Brea, USA). Cultures were grown at 30 °C with a 3 mm shaking diameter at 1200 rpm, > 85% relative humidity, an initial filling volume of 0.8 mL per well, and a starting OD_600_ of 0.1. During cultivation, backscatter (scattered light), pH, and dissolved oxygen (DO) were monitored non-invasively every 5 min. DO was maintained via a cascade control of the inlet air oxygen concentration between 21 and 70%.

Fed-batch operation utilized a feed solution containing 500 g*L^−1^ glycerol. The first exponential fed-batch phase was performed with a feed profile of 2.97 µL*h^−1^ * e^0.14*t^. This was followed by a constant feeding phase with rates of 0–40% of the maximum glycerol uptake rate previously determined at the time point of transition to this phase. The maximum glycerol uptake rate obtained from previous bioreactor experiments was used to calculate the corresponding feed rates, enabling glycerol feeding at defined fractions of 0–40% in microscale cultivations.

Optical pH monitoring allowed single-sided control to pH 6 starting 10 h after inoculation. Titration was performed using 0.5% (v*v^−1^) ammonium hydroxide with PI control settings of P = 2, I = 1, and a deadband of 0.05. To prevent clogging of the microfluidic channels, all feed solutions were sterile-filtered through 0.2 µm cellulose acetate syringe filters (DIA-Nielsen, Düren, Germany). Biomass signals were baseline-corrected by subtracting the mean of the first five measurement cycles.

### Cell dry weight determination

To measure cell dry weight (CDW), 1.5 mL Eppendorf tubes were dried in a drying oven for 24 h, then further dried in a desiccator for another 24 h before being weighed. For sampling, 1 mL of culture was centrifuged at 21,000 $xg$ and 4 °C for 5 min (Centrifuge CT 15RE, VWR International GmbH, Darmstadt, Germany). The supernatant was frozen at − 20 °C, and the pellet was dried in a drying oven for 24 h and then in a desiccator for at least another 24 h before final weighing to calculate CDW.

### ABTS assay for peroxidase activity assessment

To measure peroxidase activity, culture supernatants were diluted 1:100 with assay buffer. (210 mM sodium acetate, pH 4.5). From this, 20 µL of the diluted sample was added to 180 µL of ABTS substrate mix. The substrate solution consisted of 19 mL of assay buffer, 1 mL of ABTS stock. (17.1 mM ABTS in 50 mM sodium acetate, pH 4.5), and 7.5 µL of 3% hydrogen peroxide. Absorbance at 405 nm was recorded over a 10-min period using a microplate reader (Infinite M200 Pro, Tecan, Männedorf, Switzerland), with readings taken approximately every 40 s. Enzymatic activity (U) was calculated using the following equation:

$$ U = \left( {\Delta A\Delta t^{ - 1} * Vtot * D} \right) / \left( {Vsample * \varepsilon * d} \right) $$

$U$ units per mL [µmol*mL^−1^*min^−1^].

$V_{tot}$ total reaction volume [mL].

$\Delta A\Delta t^{ - 1}$ change in absorption at 405 nm per time [min^−1^].

$$D$$ dilution factor.

$d$ layer thickness [cm].

$V_{{{\mathrm{sample}}}}$ sample volume [mL].

$\varepsilon$ extinction coefficient at 405 nm [36] [mL*µmol^−1^*cm^−1^].

## Results and discussion

### Reference process: methanol induction

**Fig. 1:**
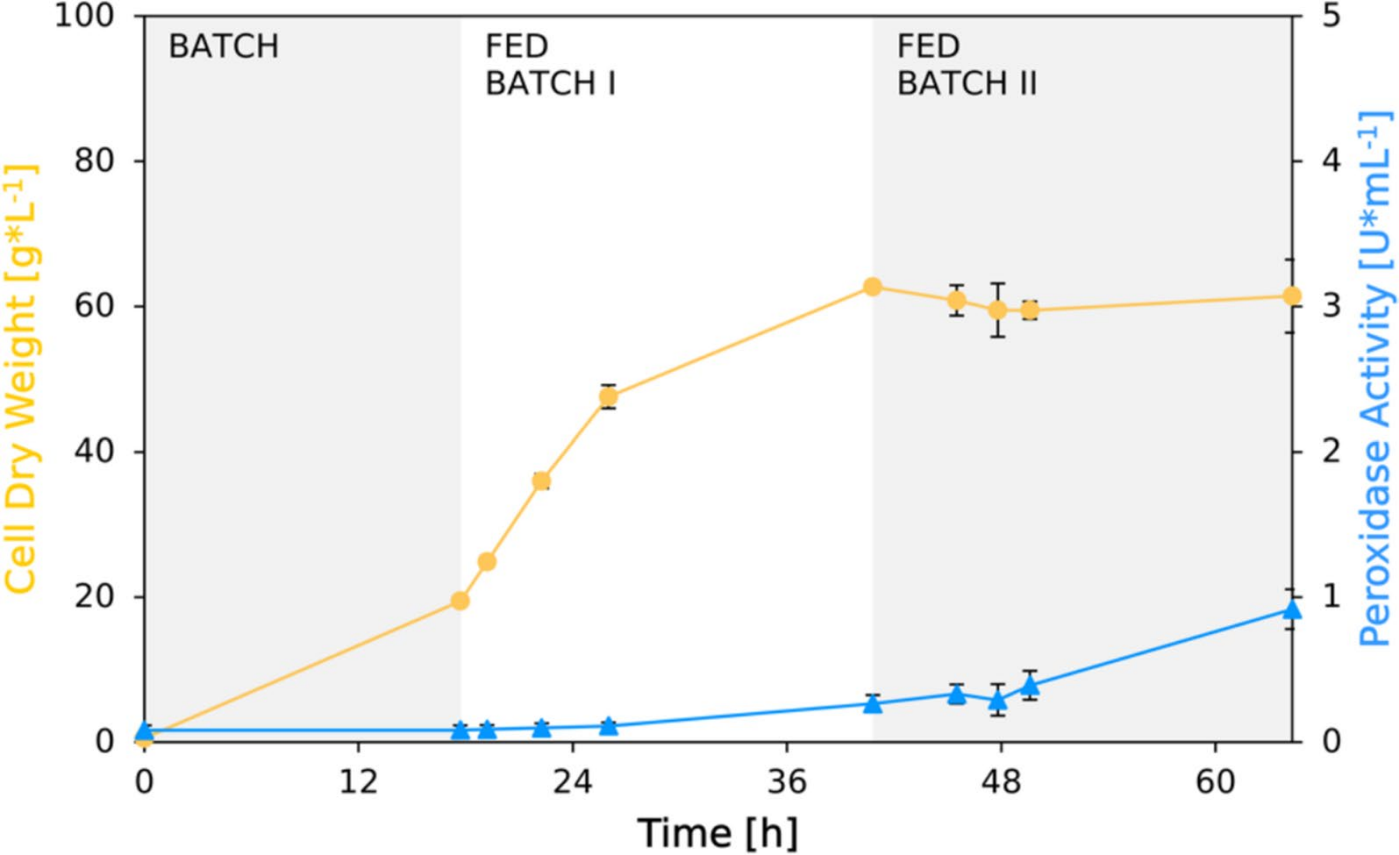
Peroxidase production process utilizing methanol induction; displayed are the cell dry weight and volumetric peroxidase activity for the three-phase bioprocess (n_technical_ = 3). The cultivation was conducted in adapted basal salt medium, at 30 °C and pH = 6. After an initial batch phase with 20 g*L^−1^ glycerol and a following constant glycerol feed of 6.25 g*h^−1^ in first fed-batch phase, the process was shifted to methanol for promoter induction at a constant feeding rate for 1.58 g*h^−1^. Figure 1 shows only one of three biological replicates with similar final activity values of 0.91–0.94 U*mL^−1^

The P_DF_ promoter implemented in the expression cassette of the utilized *K. phaffii* strain enables induction through the addition of methanol to the cultivation medium. Due to the methylotrophic nature of *K. phaffii*, methanol can be metabolized, making methanol induction a feasible process strategy. Consequently, a three-phase cultivation strategy was established. An initial glycerol batch phase (20 g*L^−1^) was followed by a constant glycerol feed of 6.25 g*h^−1^ in the first fed-batch phase to achieve sufficient biomass concentration for induction. Subsequently, a second fed-batch phase with constant methanol feed of 1.58 g*h^−1^ was applied, representing 80% of maximal methanol uptake at this process time, to induce peroxidase production (Fig. 1).

During the first two process phases, biomass increased to approximately 60 g*L^−1^ cell dry weight, while only minimal peroxidase production was observed. In the third phase, the substrate switch to methanol stopped biomass formation but initiated peroxidase production, resulting in a final volumetric peroxidase activity of 0.92 U*mL^−1^. The process demonstrated consistent performance across three biological replicates, with final peroxidase activity levels ranging from 0.91 to 0.94 U*mL^−1^. This feeding regime is comparable to previously reported methanol-based induction processes [27, 28, 37], showing robust performance and high induction strength. In addition, the P_DF_ promoter not only enables glycerol derepression as an alternative induction strategy but has also been shown to outperform the standard P_*AOX1*_ promoter by roughly a factor of 3 when induced by methanol [24].

A successful glycerol derepression as induction strategy would eliminate the need for methanol, which poses safety concerns due to its volatility and flammability, and imposes a higher oxygen demand and greater heat generation during metabolism than glycerol [30, 31]. Furthermore, the availability of glycerol as a biodiesel production sidestream underlines an additional advantage of methanol-free process strategies.

### Process adaptation: glycerol derepression

**Fig. 2:**
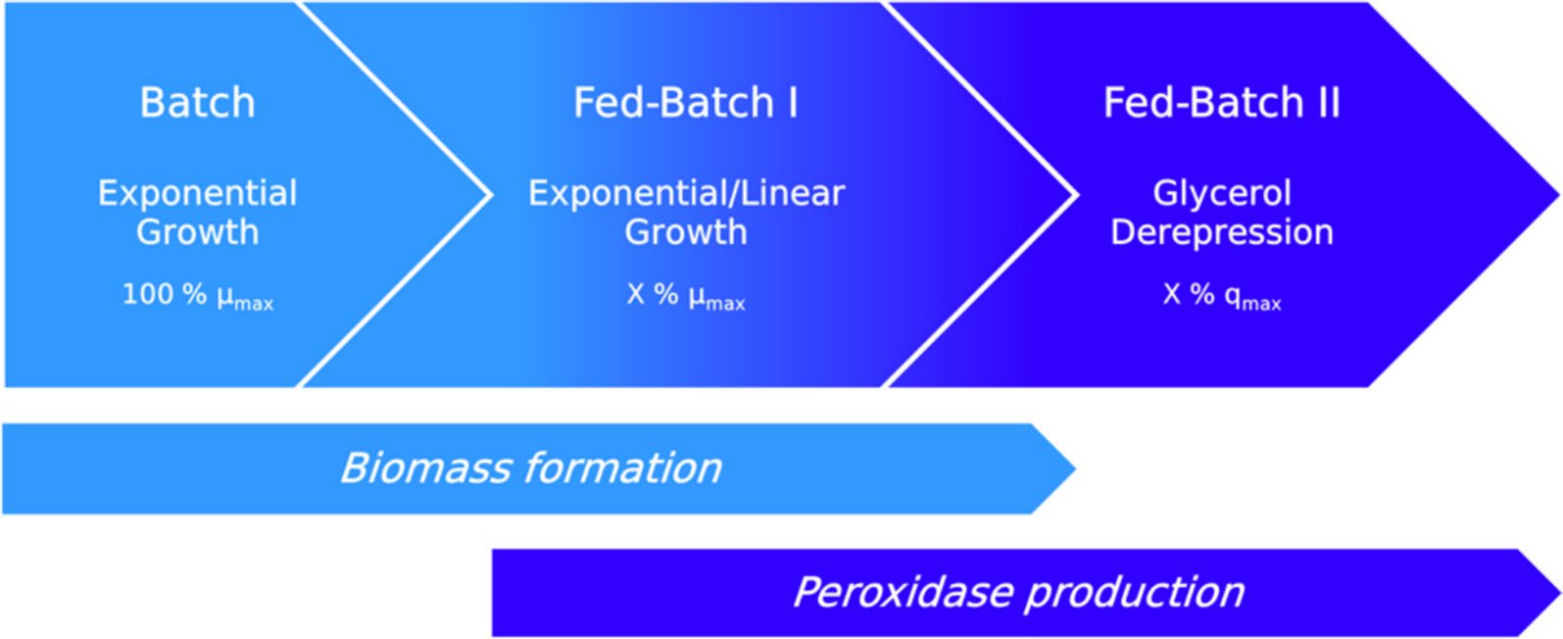
Schematic overview of the established glycerol-based process strategy; the process comprises three phases: a glycerol batch phase and a first glycerol fed-batch phase for biomass growth and induction, following a second glycerol fed-batch phase in which only reduced amounts of glycerol are being fed to induce peroxidase production

Given the advantages of a methanol-free process strategy, a solely glycerol-based process was established. A schematic overview of the process is shown in Fig. 2.

The cultivation strategy comprised three sequential phases designed to separate biomass formation from protein expression. During the initial glycerol batch phase, cells grew under carbon-excess conditions until the available glycerol was depleted. In the subsequent glycerol fed-batch phase, FB I, exponential glycerol feeding was used to further increase biomass in a controlled manner. The process was then shifted to FB II, in which lower glycerol feed rates create stronger carbon-limited conditions compared to FB I. This limitation relieved glycerol-dependent repression of the P_DF_ promoter, thereby enabling promoter derepression and peroxidase production. As a first optimization step, the feeding strategy of the initial first fed-batch phase was systematically evaluated.

### Growth rate optimization during the first fed-batch phase

**Fig. 3:**
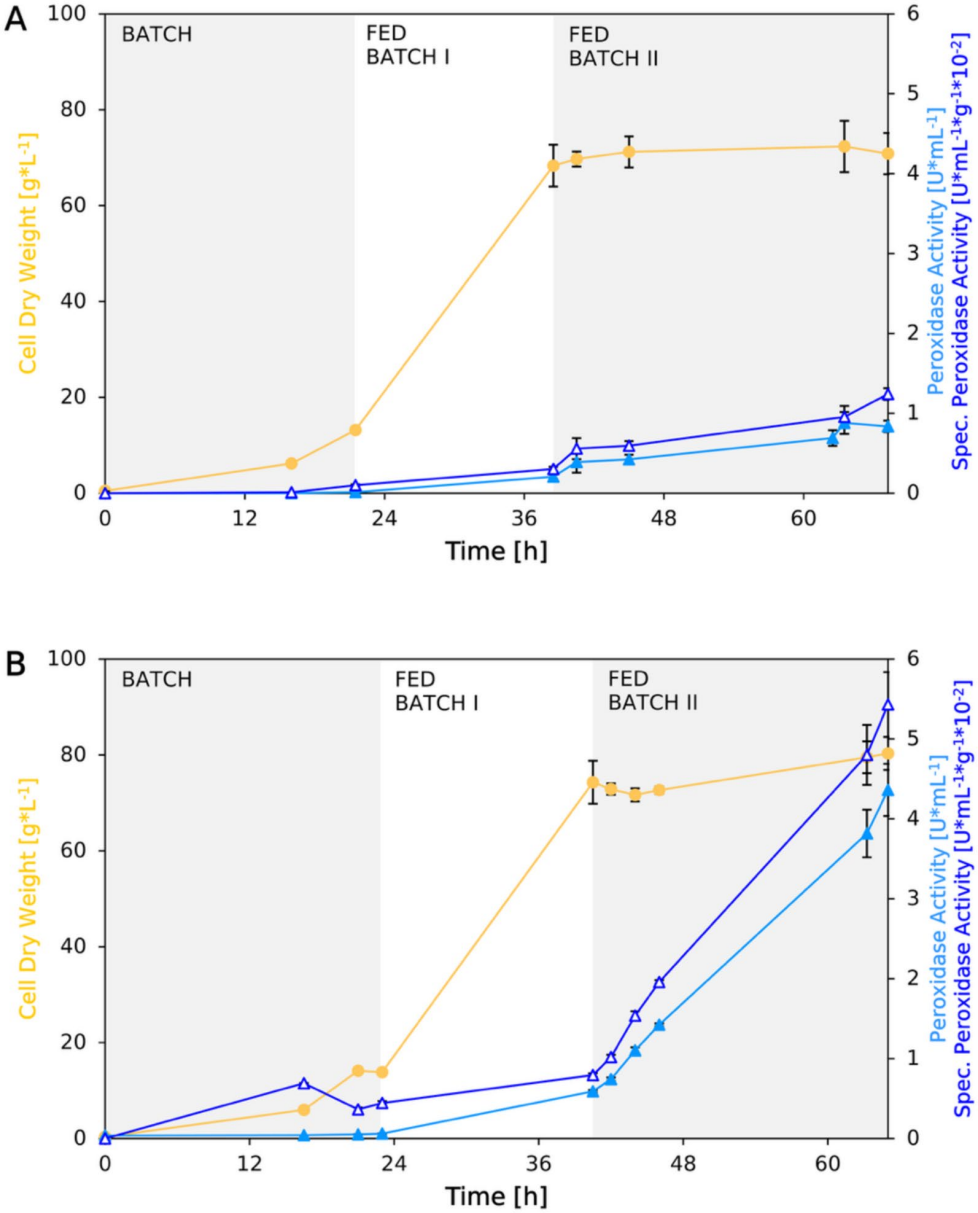
Peroxidase production process utilizing glycerol derepression; displayed are the cell dry weight, volumetric peroxidase activity and specific peroxidase activity for different process strategies during the first fed-batch phase (n_technical_ = 3). **A** Constant glycerol feeding during the first fed-batch with a feed rate of 6.25 g*h^−1^. **B** Exponential glycerol feeding at 50% of µ_max_ during the first fed-batch. The cultivations were conducted in adapted basal salt medium, at 30 °C and pH = 6. After an initial batch phase with 20 g*L^−1^ glycerol and a following constant or exponential fed-batch phase with 100 g of glycerol, metabolism was shifted to glycerol derepression representing 20% of maximal glycerol uptake at this process time

As no a priori predictions can be made regarding the optimal process strategy, different feeding regimes were evaluated with respect to key performance indicators (KPIs) such as titer, yield and productivity. As a first optimization target, the initial FB I was examined. Two feeding profiles–constant and exponential–were tested, administering the same total amount of glycerol over the same duration of the process phase. This comparison was designed to determine whether the metabolic state of cells during the growth and induction FB I influences the product formation during the subsequent production FB II with glycerol derepression at 20% of maximal glycerol uptake at this process time (Fig. 3).

The maximum growth rate µ_max_ = 0.286 h^−1^ of the *K. phaffii* peroxidase production strain was determined in bioreactor cultivation with 20 g*L^−1^ glycerol batch substrate concentration and. µ = 0.143 h^−1^ was used for the exponential feeding profile.

Biomass accumulation in FB II with the linear feeding profile (Fig. 3, A) was almost the same as with the exponential feeding profile (Fig. 3, B) reaching approximately 70 g*L^−1^ CDW at the end of FB I, with no relevant change of biomass concentration during FB II in both cases. This similarity is consistent with the fact that, despite the different temporal distribution of the feeds, the total amount of glycerol fed was equivalent, and therefore a comparable final biomass concentration is expected. On the other hand, the specific growth rate was probably different, being constant for the exponential profile but changing significantly along this fed-batch phase for the constant feeding.

Strikingly, a pronounced effect on peroxidase production was observed. The constant glycerol feeding in FB I resulted in a final peroxidase activity of 0.83 U*mL^−1^, which is comparable to the methanol-based reference process, while exponential feeding led to a final peroxidase activity of 4.36 U*mL^−1^, representing 5.2-fold improvement in final activity. Because the cell dry weight profiles were comparable across both experiments, the specific peroxidase activity showed a similar trend in both scenarios. Interestingly, FB I phase contributed a minor portion of the overall activity in both cases, but exponential feeding seemed to provide superior induction conditions for the P_DF_ promoter to enable product formation based on glycerol derepression mechanism.

Although the induction mechanism of the P_DF_ promoter is not yet fully understood, these results demonstrate that induction strength is influenced not solely by glycerol availability during FB II, but also by the preceding metabolic state of the cells in FB I. Exponential feeding maintains cells in a consistent metabolic state, here at 50% of their maximum growth rate, whereas the constant feeding profile is represented by glycerol overfeeding in the initial part of FB I and limited feeding in the later part. Nevertheless, although both feeding profiles represent glycerol-limited conditions at the end of FB I, the consistent growth conditions under exponential feeding appear to make an essential difference and benefit P_DF_ promoter induction.

A possible explanation might be the temporary accumulation of intracellular storage compounds during FB I, such as fatty acids, which may be metabolized during FB II. Since glycerol metabolism is closely linked to fatty acid metabolism, potential build-up and breakdown of storage components could play a role [38–40]. In addition to intracellular storage metabolism, extracellular metabolites generated under the different FB I feeding regimes may also contribute to P_DF_ promoter activation. Such metabolites could accumulate in the culture medium and act either as direct inducing signals or as indirect indicators of the physiological state that favors promoter derepression. Therefore, future studies should include targeted or untargeted analysis of extracellular metabolites during FB I and the transition to FB II to identify compounds potentially involved in P_DF_ promoter activation.

To further investigate, different growth rates were applied during exponential feeding in FB I and total input of 100 g glycerol per reactor was maintained to facilitate fair comparison of conditions in terms of substrate supplied. All conditions led to final biomass concentration of approx. 80 g*L^−1^ cell dry weight, stressing that observed effects were not caused by differing biomass concentrations but rather regulatory effects.

**Fig. 4:**
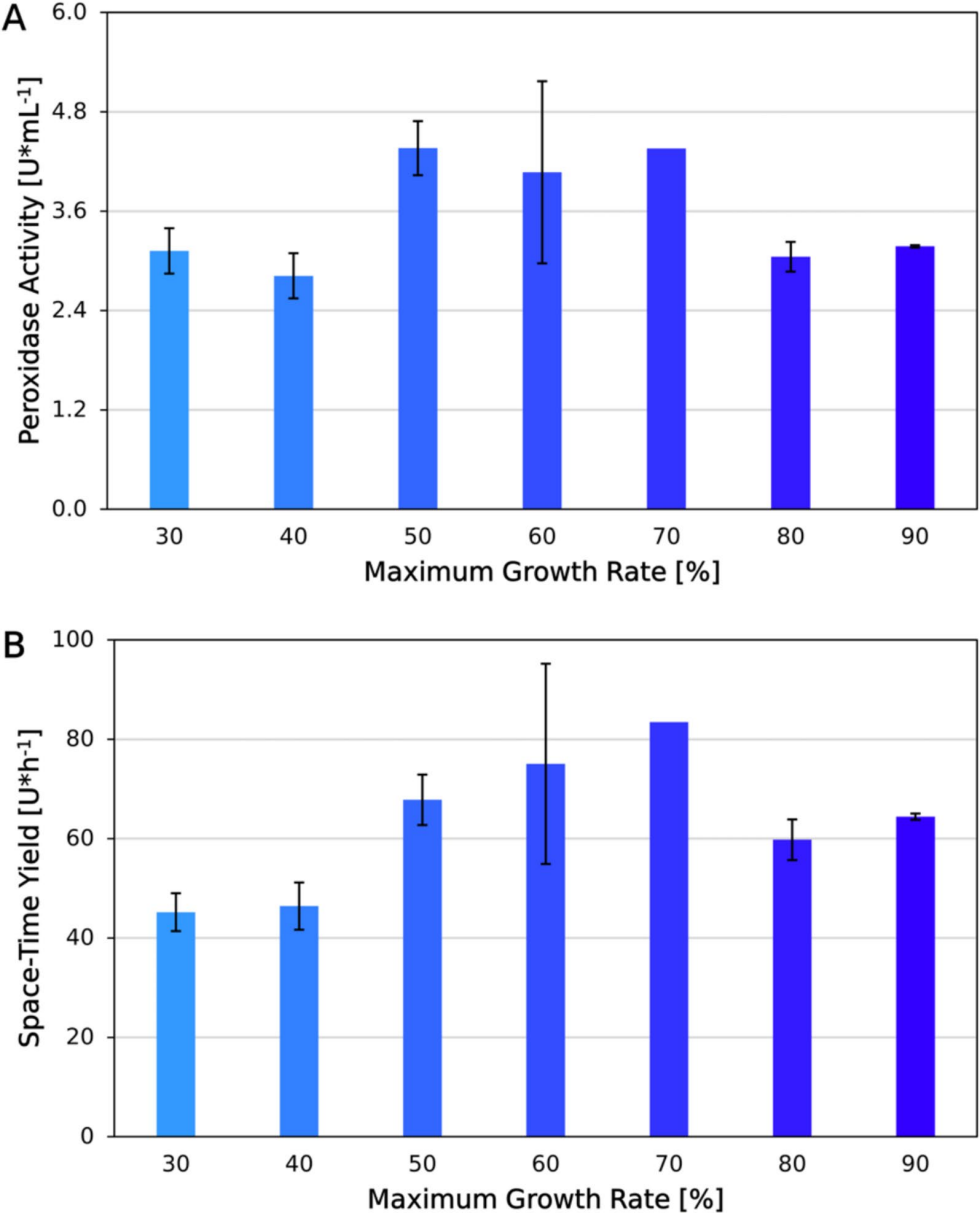
Variation of the growth rate during the first fed-batch phase results in varying process performance. Displayed are the final peroxidase activity (**A**) and the space–time yield (**B**) for different growth rates during the first fed-batch phase (n_biological_ = 2, except for µ = 70% µ_max_). The cultivation was conducted in adapted basal salt medium, at 30 °C and pH = 6. After an initial batch phase with 20 g*L^−1^ glycerol and a following exponential fed-batch phase with 100 g of glycerol, metabolism was shifted to glycerol derepression for promoter induction at a constant feeding rate

Exponential glycerol feeds in FB I corresponding to growth rates between 30 and 90% of µ_max_ were tested and the FB II production phase was kept constant with glycerol derepression at 20% of maximal glycerol uptake at this process time. The resulting volumetric peroxidase activities and space–time yields (STY) are summarized in Fig. 4.

Moderate growth rates (50–70% of µ_max_) in FB II led to ~ 40% higher peroxidase activity compared to both lower and higher exponential growth rates (Fig. 4, A). These rates also delivered superior space–time yields, balancing elevated productivity with acceptable process durations. Conversely, higher growth rates decreased product activity and increased heat generation, which can negatively affect product stability and increase process costs (Fig. 4, B) [41]. Considering all factors, a growth rate of 50% of µ_max_ was selected as the optimal condition for FB I.

Because regulation of the P_DF_ promoter under glycerol limitation is still poorly characterized, the observed induction differences should be interpreted in relation to specific growth rate, carbon catabolite repression, and cellular physiology. Glycerol excess likely maintains promoter repression, whereas glycerol limitation during FB II supports derepression. However, the different outcomes after exponential and constant feeding indicate that P_DF_ activity is not determined solely by glycerol availability during induction, but also by the metabolic state established during FB I.

Higher specific growth rates may additionally increase metabolic burden and secretory stress, particularly for secreted enzymes, thereby limiting productive expression despite enhanced biomass formation. Because glycerol metabolism might be linked to lipid and fatty acid metabolism, changes in storage lipid formation, lipid turnover, or membrane composition may also affect secretion efficiency. Compared with classical system such as P_AOX1_, which is mainly methanol inducible, and P_GAP_, which is constitutively active during growth on a suitable carbon source, P_DF_ appears to combine carbon-source-dependent repression, derepression under glycerol limitation, and optional methanol inducibility. Further studies combining defined growth-rate conditions with intra- and extracellular metabolite profiling could help clarify the metabolic signals involved in P_DF_ promoter activation.

### Derepression optimization during the second fed-batch phase

**Fig. 5:**
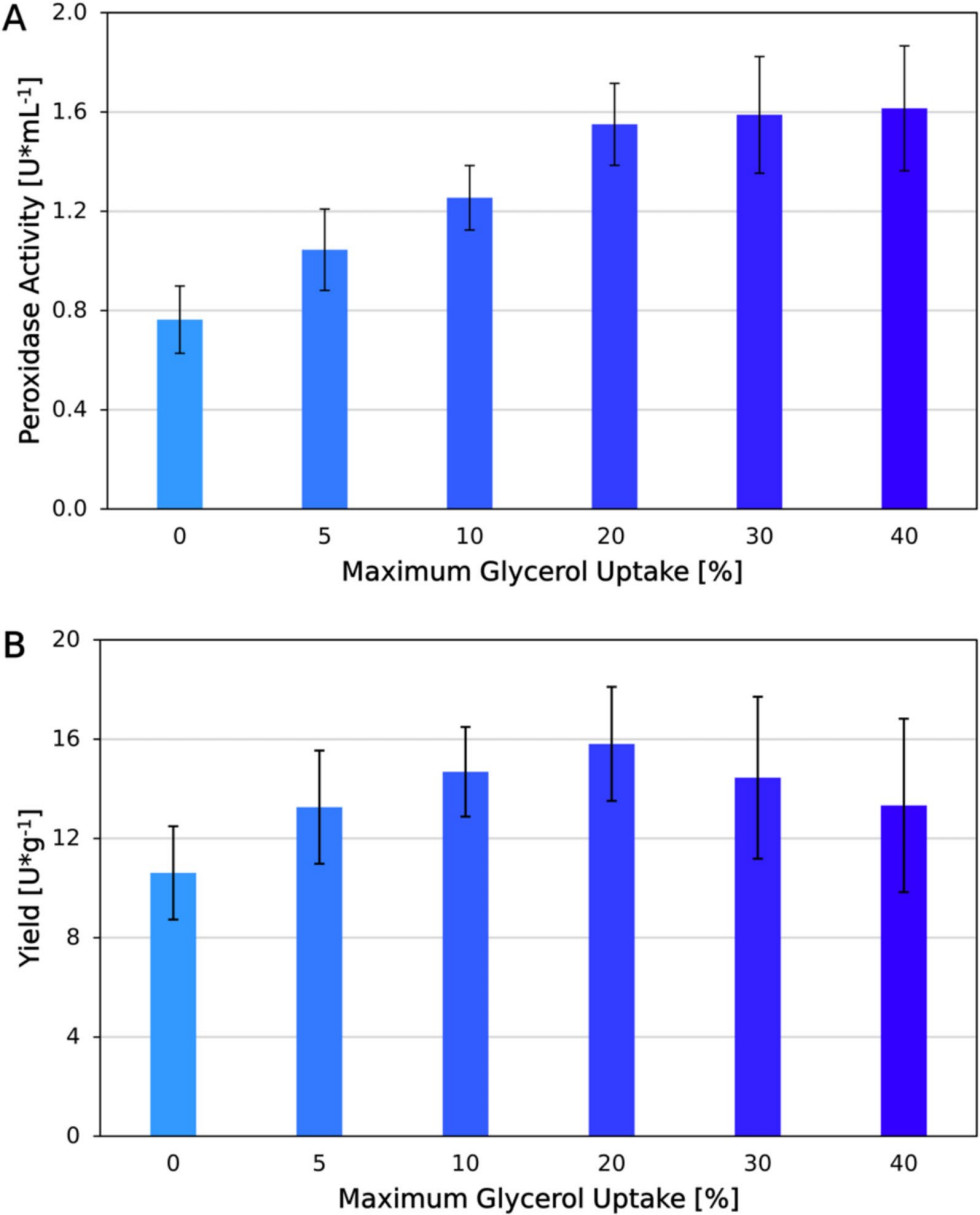
Variation of the feed rate during the second fed-batch phase results in varying process performance. Displayed are the final peroxidase activity (**A**) and the yield (**B**) for different maximum glycerol uptake rates during the second fed-batch phase (n_biological_ > 3). The cultivation was conducted in adapted basal salt medium, at 30 °C and pH = 6. After an initial batch phase with 20 g*L^−1^ glycerol and a following exponential fed-batch phase at 50% of µ_max_, metabolism was shifted to glycerol derepression for promoter induction at a constant feeding. The cultivations were performed in the BioLectorXT to enable higher throughput and therefore a higher number of replicates

Following the optimization of the first fed-batch phase (FB I), the second fed-batch (FB II) during which peroxidase production is induced via glycerol derepression was further optimized. To define an appropriate range of feeding rates, the maximum glycerol uptake capacity of the culture at the onset of FB II was determined. For this, a 3 g pulse of pure glycerol was added immediately after the conclusion of FB I, and the dissolved oxygen (DO) signal was monitored with the DO cascade deactivated. The time required to metabolize the glycerol pulse was recorded at 18.7 min, corresponding to an apparent maximum glycerol uptake rate of 9.62 g*h^−1^ (0.128 g_S_*g_X_^−1^*h^−1^). Based on this value, glycerol feeding strategies during FB II were tested in the BioLectorXT system at rates ranging from 0 to 40% of this maximum uptake rate (Fig. 5).

A clear increase in peroxidase production was observed with increasing glycerol feeding rates from 5 up to 20% of the maximum uptake rate, followed by constant values for the peroxidase activity up to 40% (Fig. 5, A). Please note that 0% represents no feed addition in FB II. While the final peroxidase titers remained constant at feeding rates higher than 20%, consequently the overall process yield declined due to excess substrate input without corresponding product formation (Fig. 5, B). These results indicate that limiting glycerol feeding provides derepressed promoter activity and peroxidase production. However, increasing glycerol beyond 20% of such threshold seems to offer no additional benefit for product formation, but reduces product yield. Thus, 20% of the maximum glycerol uptake rate was identified as the optimal derepression feeding strategy for P_DF_ promoter induction in FB II.

**Fig. 6:**
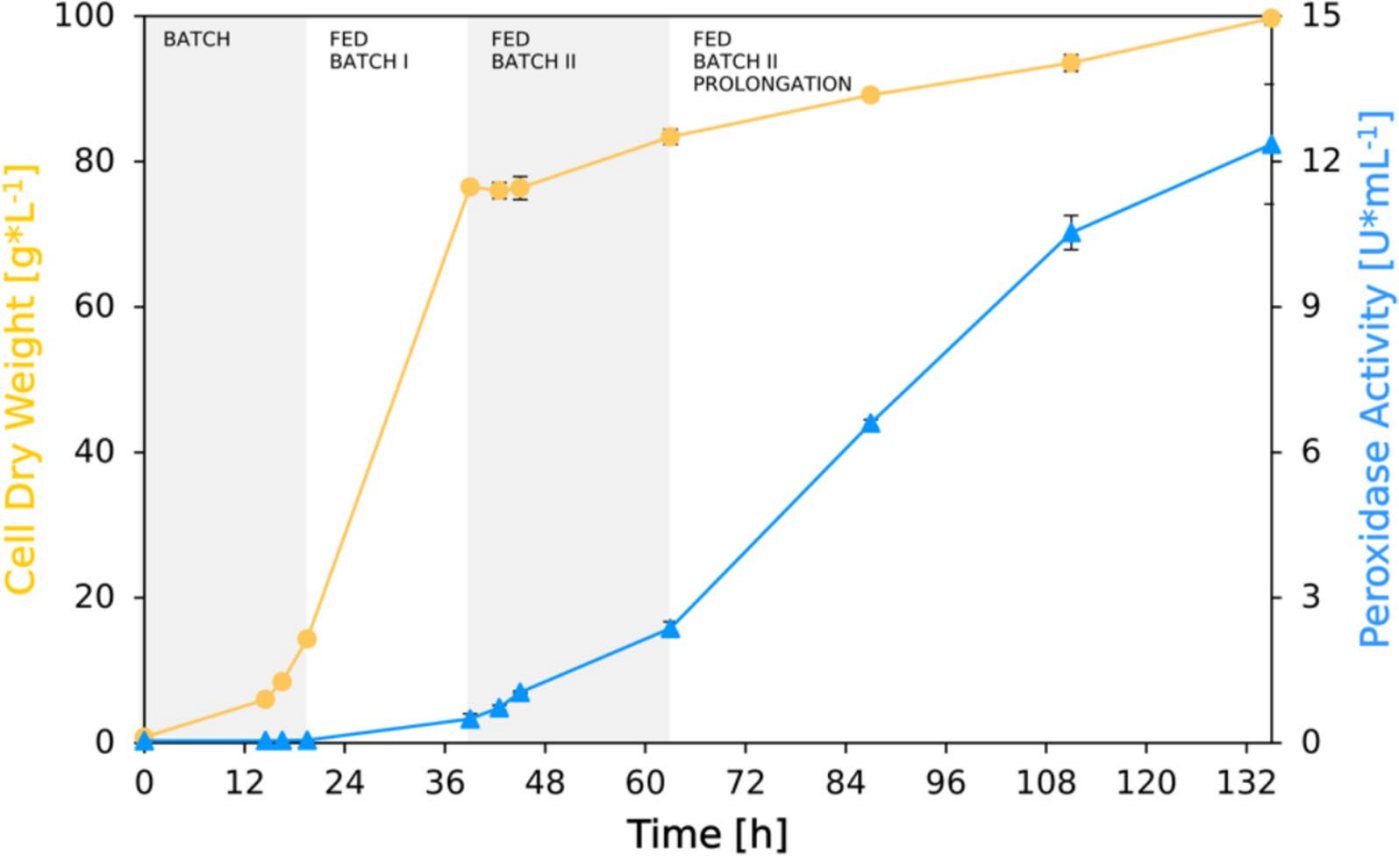
Prolongation of the second fed-batch does not show a decrease of peroxidase production rate throughout the cultivation; displayed are the cell dry weight and volumetric peroxidase activity for different process strategies during the first fed-batch phase (n_technical_ = 3). The cultivations were conducted in adapted basal salt medium, at 30 °C and pH = 6. After an initial batch phase with 20 g*L^−1^ glycerol and a following exponential fed-batch at 50% of µ_max_ with 100 g of glycerol, metabolism was shifted to glycerol derepression

Subsequently, the duration of FB II was investigated. In prior experiments (Fig. 3 B), peroxidase activity continued to increase steadily throughout the 24-h derepression and production phase FB II, suggesting that prolonged cultivation could further enhance peroxidase concentration. Therefore, the duration of FB II was extended from 24 to 96 h (Fig. 6).

The extended process revealed sustained high peroxidase production throughout the derepression phase and was highly comparable to the previous experiment using same conditions in phase FB I and FB II, culminating in a final activity of 12.36 U*mL^−1^. Notably, no decline in production rate was observed until the final 24 h and no signs of product degradation were detected. As a result, the overall space–time yield was significantly improved, increasing from 0.067 U*mL^−1^*h^−1^ to 0.081 U*mL^−1^*h^−1^. The overall yield was increased from 28.36 U*g^−1^ to 46.31 U*g^−1^.

### Crude glycerol as an alternative carbon source

Crude glycerol, a by-product of biodiesel production, constitutes an industrial sidestream with significant valorization potential. Its use not only enhances the circularity of the biodiesel value chain, but also contributes to reducing overall production costs, thereby increasing the economic viability of biodiesel as a renewable fuel. As such, the utilization of crude glycerol aligns with the goals of a more sustainable and resource-efficient economy.

**Fig. 7:**
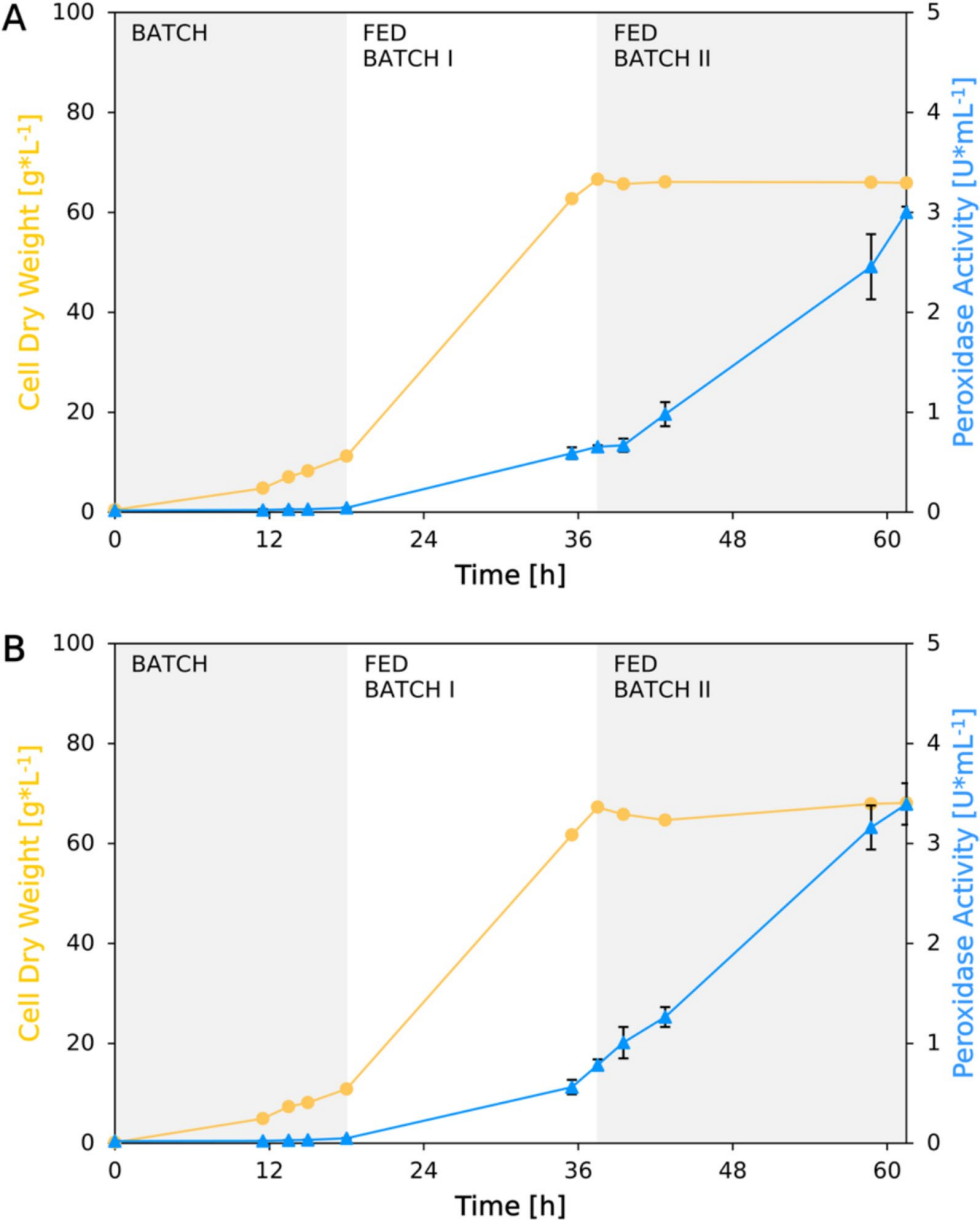
Comparison of crude and purified glycerol as the carbon source for the developed process strategy; displayed are the cell dry weight and volumetric peroxidase activity (n_technical_ = 3). **A** Purified glycerol as substrate for the whole process. **B** Crude glycerol as substrate for the whole process. The cultivations were conducted in adapted basal salt medium, at 30 °C and pH = 6. After an initial batch phase with 10 g*L^−1^ glycerol and a following exponential fed-batch at 50% of µ_max_ with 110 g of glycerol, metabolism was shifted to glycerol derepression

To evaluate the feasibility of using crude glycerol in the established production process, comparative experiments were performed using both purified and crude glycerol. The crude glycerol utilized in this study was sourced from Cargill’s Bioro refinery in Ghent, Belgium. It has a purity of 80% and contains 10% sodium chloride, 4% of organic matter and 0.5% of methanol as the major impurities (Fig. 7).

Crude glycerol did not lead to a decrease in process performance; on the contrary, it resulted in a slight increase in final peroxidase titer. The process utilizing crude glycerol achieved a final activity of 3.4 U*mL^−1^, compared to 3.0 U*mL^−1^ with purified glycerol. These results demonstrate the feasibility of crude glycerol as a viable substrate alternative and highlight the robustness of *K. phaffii* against common impurities present in crude glycerol, such as residual methanol, elevated sodium chloride concentrations, and organic contaminants. It can be speculated that the minor methanol content might have played a positive role in the P_DF_ promoter activation.

## Conclusions

This study aimed to establish and optimize a three-phase bioprocess to produce peroxidases in.

*K. phaffii* under the control of the P_DF_ promoter. The process comprises an initial batch phase, a first fed-batch phase for biomass accumulation, and a second fed-batch phase for promoter induction via glycerol derepression. The bioprocess development campaign focused on maximizing key performance indicators such as titer, yield, and space–time yield.

The first fed-batch phase was mainly intended for biomass growth and induction and was optimized regarding feeding strategy and feed rates. Exponential feeding significantly outperformed constant feeding leading to improvements of up to 420% in KPIs. Following optimization of the specific growth rate revealed that moderate growth rates yielded the best results. 50% of µ_max_ was chosen as the optimal process condition and was a prerequisite for high productivity resulting in final peroxidase titers of 4.36 U*mL^−1^.

The second fed-batch phase was optimized regarding feed rate and product formation. Feed rates equivalent to 0–40% of q_Smax_ were evaluated. It was observed that above a threshold of 20% of q_Smax_, final peroxidase titer did not improve further. Extending the second fed-batch phase revealed that productivity did not diminish for up to 72 h, with a slight decrease only in the final 24 h of the 96-h phase. The fully optimized process achieved a final titer of 12.36 U*mL−1, a yield of 46.31 U*g−1 and a space–time yield of 0.081 U*mL^−1^*h^−1^.

Process robustness was further demonstrated using crude glycerol as a carbon source. Despite the presence of impurities such as methanol, sodium chloride and organic matter, no decrease in performance was observed. On the contrary, a 13% increase in process performance was achieved which might have been influenced by residual methanol.

In summary, this study presents a robust and efficient bioprocess for the production of peroxidases using renewable feedstocks. Key performance indicators were improved by around 13-fold, supporting the feasibility of this process for industrial application. The developed process is a valuable step toward sustainable production of peroxidases, enabling the substitution of cobalt-based siccatives in polyester resin curing and therefore contributing to greener manufacturing practices.

## Data Availability

Experimental raw data and datasets used and/or analyzed during the current study are available from the corresponding author on reasonable request.

## Abbreviations

°C: Degrees Celsius
ABTS: 2,2′-azino-bis(3-ethylbenzothiazoline-6-sulfonic acid)
cm: Centimeter
g: Gram
h: Hour
K. phaffii: *Komagataella phaffii*
L: Liter
mg: Milligram
min: Minute
mL: Milliliter
MTP: Microtiter plate
nm: Nanometer
rpm: Revolutions per minute
sec: Second
U: Unit
g: Earth acceleration
µL: Microliter
µmol: Micromol
